# Aroused and Impulsive Effects of Colour Stimuli on Lateral and Logical Abilities

**DOI:** 10.3390/bs11020024

**Published:** 2021-02-07

**Authors:** Guobin Xia, Muzi Li, Philip Henry, Stephen Westland, Francisco Queiroz, Qiwei Peng, Luwen Yu

**Affiliations:** 1 School of Design, University of Leeds, Leeds LS2 9JT, UK; P.M.Henry@leeds.ac.uk (P.H.); s.westland@leeds.ac.uk (S.W.); f.queiroz@leeds.ac.uk (F.Q.); ml14l2y@leeds.ac.uk (L.Y.); 2 School of Media, Harbin Normal University, Harbin 150080, China; woodlee.ca@gmail.com; 3 Department of Informatics, University of Sussex, Brighton BN1 4GE, UK; qp24@sussex.ac.uk

**Keywords:** colour psychology, arousal, impulsiveness, cognitive abilities

## Abstract

The purpose of this study was to explore the influence of environmental colour on people’s lateral and logical abilities. This was done by evaluating study participants’ response time and error rate when completing six types of psychometric tests that were performed in various hue backgrounds on a computer. To maximise the colour stimulation provided by the monitor, the experiment was carried out in a dark laboratory. Analysis of participants’ response time and error rate showed that different colours could significantly influence arousal and impulsiveness, which suggests that colour has indirect impacts on cognitive abilities. Further analysis revealed that different colours had various effects depending on the type of psychometric test given. These findings suggest that future research on environmental design should consider how to effectively use colour to impact people’s performance and behaviour.

## 1. Introduction

The study evaluated the influence of colour stimuli on people’s cognitive abilities with particular focus on logical and lateral abilities. The human brain divides into two distinct cerebral hemispheres, and each of them tends to lateralise and specialise in different cognitive abilities [1,2,3]. Notably, the right hemisphere is responsible for lateral abilities (i.e., creative thinking, imagination, holistic perception and emotional thought), while the left hemisphere is in charge of logical abilities (i.e., analytical thought, detail-oriented perception, ordered sequencing, rational thought, and math/science) [4,5,6,7]. Colour and light as a ubiquitous perceptual stimulus have been manifested in the previous studies in optimistically affecting people’s cognitive functions [8], human perceptions [9], psychological and emotional reactions and ultimately [10,11], behavioural intentions [12]. However, research investigating the influence of colour stimuli on people’s lateral and logical abilities is limited. Questions that this study deems significant and attempts to answer are (1) whether colours could influence people’s cognitive abilities, and (2) how?

There is a rich history of studies that relates to how environmental colours can affect people’s behaviours and performance. For instance, Elliot, et al. [13] investigated the connection between colour and human psychological reactions with particular focus on red and performance attainment. Results of their work found a clear link between colours and emotions through various observed behavioural (i.e., task choice) and psychophysiological (i.e., cortical activation) reactions. The study by Elliot, et al. [13] was impressive in its contribution to illustrate that colour can act as a subtle environmental cue that has essential impacts on people’s behaviours. Yildirim, et al. [14] studied the effects of three different colours (cream, blue, and pink) on the interior wall surfaces of classrooms on the perceived performance of male students. They observed that students felt more positive in spaces with blue walls compared to cream and pink coloured spaces. It is suggested that the effective use of colours in the design of classrooms do have significant impacts on students’ perceptual performances. Interestingly, some studies carried out explored the intensity lighting impacts have on people’s electroencephalogram (EEG) power [15,16]. Results have demonstrated the stimulus of short and long wavelength light on people’s alpha, theta, and beta power, suggesting that coloured light can promote acute alertness and improve performance on tasks requiring sustained attention.

The impacts of colour inducements on cognitive performance can also be observed in textile design. Significant contributions have been made by Hill and Barton [17], Ilie, et al. [18] and Attrill, et al. [19] in several experiments demonstrating that red relative to blue clothes have significantly higher opportunities to win in competition or matches. Apart from findings regarding red effects, other researchers additionally reported judokas that wear blue might carry a better performance compared with those wearing white [20,21].

**Figure 1:**
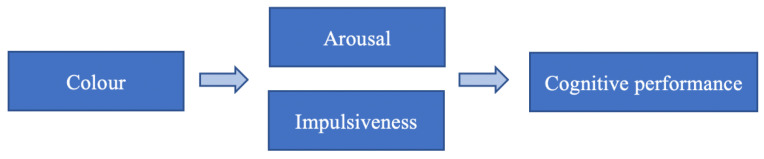
The relationship between the impacts of colour on people’s arousal and impulsive level and cognitive performance.

Other studies indicate that people’s emotions and performance can be induced by specific colours [11,22,23,24,25,26], and this indication parallels on the relationship between the impacts of colour on people’s arousal and impulsiveness (Figure 1).

Arousal refers to the physiological and psychological state of being awake. It is relatively crucial in regulating the psychological experience of attention, alertness, information processing (decision-making or judgments), emotions, memory and consciousness [27,28,29], that dimension ranges from deactivation (i.e., calm) to activation (i.e., stress or happiness) [30]. One crucial theory that attempts to explain the empirical relationship between emotional arousal and performance is the U-shaped relationship, initially established by Yerkes and Dodson, and was known as Yerkes–Dodson Law [31]. Specifically, Yerkes–Dodson Law states that raised levels of arousal can enhance performance up to a certain point; however, if beyond the optimum, increased level of arousal is followed by declines in performance [32]. For example, an optimal level of stress before an exam can increase people’s attention on the test and retain the knowledge that you have studied. In contrast, excessive test anxiety can weaken people’s ability to focus and make it more challenging to remember precise answers. Drawing on investigations of the core design elements of colour and light, generally, researchers posited that arousal difference effects could be observed and that the red end of the spectrum increased arousal and the blue reduced arousal [33,34]. Specifically, Greene, et al. [35] explored the connections among hue, arousal and boredom. In their study, a total of 140 undergraduate students (70 males, 70 females) were invited to sit in carrels and exposed to side panels painted either light blue, blue, pink, red, orange, white, brown, green, yellow, or grey. The experiment evaluates students’ aroused level by exploiting Russell and Mehrabian [32] Emotional Response Scale (ERS), Griffitt [36] Personal Feelings Scale (PFS), and Russell and Pratt [37] Affective Quality of Place Scale (AQPS). Their findings show that self-reported arousal and evaluations of the environment were higher with the yellow stimulus than in the other coloured stimulations. Collectively, the work carried out by Greene and colleagues illustrated the potential of colour stimulus as an aroused effect trigger even employing the coloured inducement in less immersive conditions.

Nevertheless, the experiment by Greene and colleagues has some problems. Firstly, they failed to manage the brightness of the colour stimulus when studying the hue influence. Moreover, when participants look at the painted side panels, they must not view a single colour, but a combination of the colour with a background colour, even though one consciously attempts to recognize only one of them. Furthermore, the results of the subjective measure are questionable, as people may mix their feelings before and after each experiment session. However, despite various methods applied in order to measure colour influence on arousal, previous insights into measuring approaches can be generally classified into three types: self-reporting methods (i.e., verbal scales), psychophysical methods (i.e., paper-folding, cognitive tasks), and psychophysiological methods (i.e., GSR, EEG, heart rate) [38,39,40,41].

Impulsiveness is defined as a behavioural ability to respond quickly and without mental reflection, which is essentially associated with the control of thoughts and behaviour [41]. It is well documented in the literature that colour can influence human perceptions and behaviours [17,21]. However, research into the measure of colour on impulsivity is relatively limited but can be generally categorized into four categories: self-report measure, behavioural measure, psychophysiological measure, everyday life experiences measure. For instance, Zentall, et al. [42] used colour stimulation with psychophysical methods to test the impulsivity of attention-problem adolescents. They compared participants’ performance through the Matching Familiar Figures Test (MFFT) between “black and white” and “colourful” patterns. Their results showed that participants were less impulsive with colourful patterns in terms of the reduced error rate of MFFT. Wang, et al. [43] conducted two psychophysical experiments to investigate the effects of environmental colour on impulsive buying behaviour. Their results revealed that environmental colour (blue vs. red) could stimulate people’s impulsive buying behaviour. To be specific, they also observed that participants who were exposed to the blue environment had higher impulsive buying intent than those exposed to the red environment. Sevda, et al. [44] explored the relationship between colour preferences and impulsive behaviour by using Beck Anxiety (BAI), Beck Depression (BDI) and Barrat Impulsivity Scales (BIS). They found colour preference is related to impulsivity. Ciccone [45] used personality, behavioural and neurological methods to study the effect of coloured environments on impulsivity in his PhD thesis, and his results conflicted with conventional opinion that long wavelength (i.e., red light) lights are encouraging and short wavelength lights (i.e., blue light) are calming. A study by Duan, Rhodes and Cheung [26] used a behavioural measure method to examine hue and found that it can have distinct impacts on impulsiveness and arousal, in which the hue seemed to have a greater impact on arousal than impulsiveness. To be specific, their findings revealed that orange and purple can influence people to exhibit a high-aroused state, while yellow leads to the least aroused state. Interestingly, in Duan, Rhodes and Cheung [26], a theoretical framework developed from the Salkind and Wright [46] integrated model was proposed to illuminate both impulsiveness and arousal based on the error-speed theory, which also can be utilized to explain the colour influence on people’s cognitive abilities in this study.

Collectively, many studies have demonstrated that colour can affect performance and behaviours but how do the effects occur on the lateral and logical abilities? Studies reviewed above help to inform the hypothesis that colour can have aroused and impulsive effects on people’s lateral and logical abilities. For a better understanding, and proliferating the potential of colour, the originality of this work builds on previous insights but goes further to develop new knowledge regarding the effective use of the colour design in triggering people’s logical and lateral functions. Psychological experiments have been carried out to study the impacts of colour on people’s aroused and impulsive level to validate the hypothesis, which is an indirect approach to validate the colour impacts on people’s lateral and logical abilities.

## 2. Methods

### 2.1. Colour Conditions

The six colour patches and an equally luminous reference white colour (used as a control) were selected from an Adobe HSB colour system based on previous research by Eysenck [47], Yu, et al. [48], Singh [49], Yu, et al. [50], and Duan, Rhodes and Cheung [26]. These colours were used as the background colour for a series of questions and adjusted to have a similar lightness and chroma based on the CIELAB values displayed on the monitor measured by the X-rite i1 Pro in dark laboratory settings (see Table A1 in Appendix A).

### 2.2. Psychometric Tests

Six types of psychometric test were utilised for measuring the participants’ logical ability (logic rule test, mathematics sequence test), lateral ability (spatial structure test, rotation test) and detail ability (odd one out, same detail test) (see Table A2). For each type of test, there were seven questions and each of these seven questions was assigned a different coloured background. This led to there being 42 questions in total (6 types of test x 7 coloured backgrounds) and each participant was asked to answer all 42 questions. The colours of the backgrounds and the orders of presentation of the questions were randomised (for each participant). However, within each test, each participant was presented with a question with each of the seven coloured backgrounds. Note, however, that for different participants the coloured backgrounds assigned to the questions within a test were different. The purpose of this is to ensure that if one of the questions, for example, was slightly more difficult than another then it would be equally likely to have any of the backgrounds for a particular participant and would remove bias.

Response time and error rate were the two main data gathered from the experiment. In the Results section, these measurements will be used to estimate participants’ aroused and impulsive levels which will be used as an indirect approach to understanding how colour impacts on people’s lateral and logical abilities.

### 2.3. Participants

A total of 21 participants (aged 20–25 years old, 10 males and 11 females) were recruited for the psychological experiment. To avoid culture effects and the possibility that some participants might be more logical in their approach, all participants were Chinese undergraduate students from the School of Media with similar academic backgrounds (animation studies).

### 2.4. Experimental Procedure

**Figure 2:**
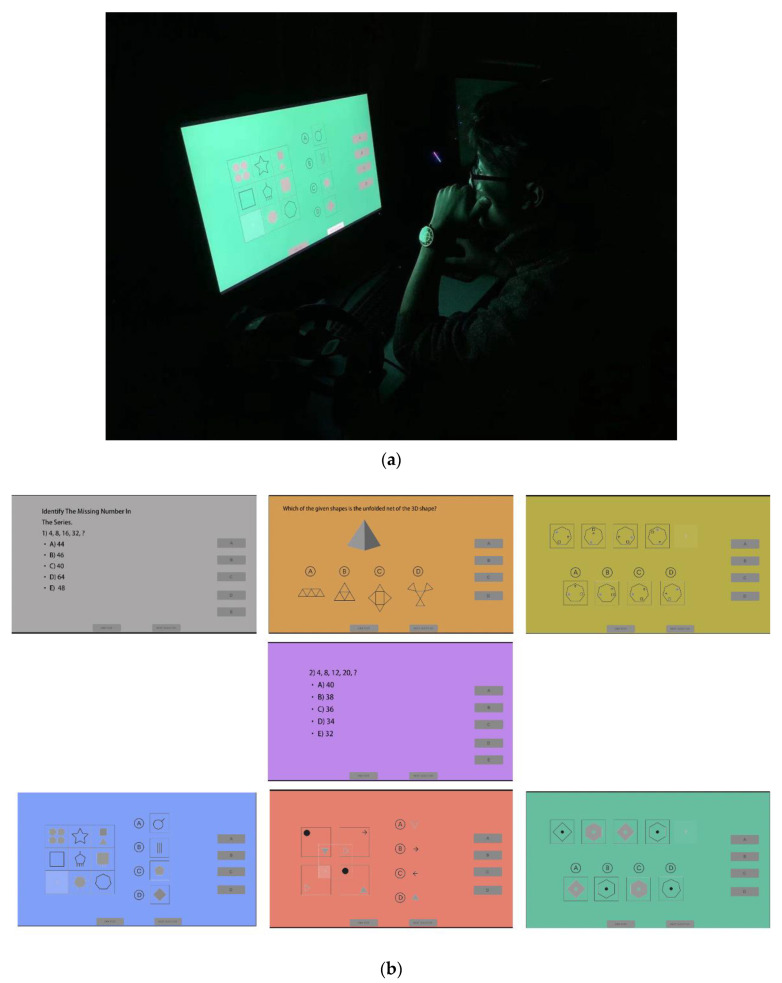
Examples of the experimental setup: (**a**) Individual participant using the mouse with the green background condition; (**b**) An example of each of the 7 coloured backgrounds used. Source: Authors.

The experiment was carried out in a dark room with each participant on their own. All participants were required to complete the Ishihara Colour Vision Test before entering the room to ensure that they had normal colour vision. After passing the test, they were asked to read the instructions concerning the entire experimental procedure. Next, a sample test including each type of psychometric test was introduced to familiarise participants with the tests before launching the formal experiment. Participants were asked to focus on the reference white background picture for five minutes to adapt to the experimental lighting conditions. The main experiment started five minutes after they had adapted to the experimental conditions. Each participant spent about 40 min to complete the main experiment. Individual participants were seated in front of a monitor and were asked to choose the right answer for each question as quickly and as accurately as possible by using a mouse (see Figure 2a,b). The monitor used in the experiment had an aspect ratio of 16:9.

## 3. Results

### 3.1. General Trend

**Figure 3:**
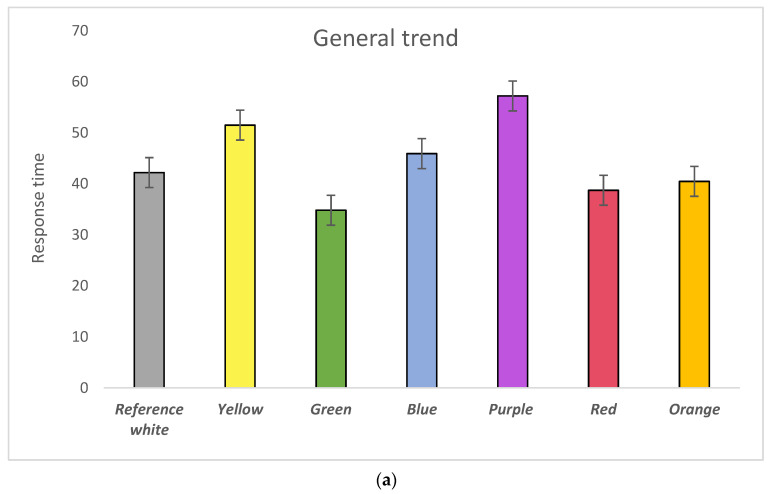

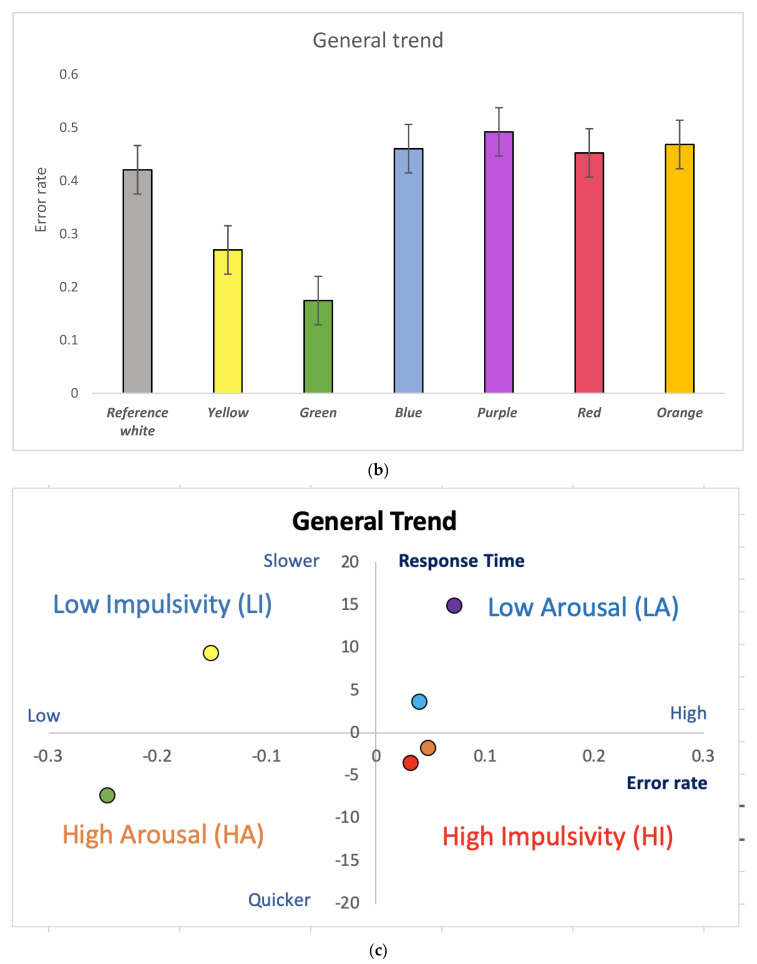
(**a**) General trend of response time by background colours; (**b**) General trend of error rate by background colours; (**c**) Colour impacts on general performance visualised in the Error-Speed space. The bars represent mean changes, while the error bars are the standard error of the mean across individual participants.

Statistical analysis was performed using Statistical Product and Service Solutions (SPSS, Armonk, NU, USA) software. Figure 3a,b shows the mean scores for response time and error rate pooled over all six types of test in completing psychological tasks. The green background gave both the fastest response and lowest error rate. A multivariate analysis of variance (MANOVA) was conducted to show the statistical significance of colour backgrounding, participants’ impulsiveness and arousal can be defined as High Arousal (HA), faster reactions and lower error rate; Low Arousal (LA), slower reactions and higher error rate; High Impulsiveness (HI), shorter response time and higher error rate; and Low Impulsiveness (LI) longer response time and lower error rate (all compared with the mean).

As for the response time (Figure 3a), participants performed faster with the reference white than the purple background (*p* = 0.032). In addition, their response time with the red (*p* = 0.008) and orange (*p* = 0.017) was shown to perform faster than the purple background. Furthermore, participants performed significantly faster with the green background than the purple (*p* = 0.001), and yellow (*p* = 0.017) backgrounds.

With regard to the error rate (Figure 3b), participants with the green background were shown to make significantly fewer errors compared with participants with the purple (*p* = 0.000), orange (*p* = 0.000), blue (*p* = 0.000) and also the reference white (*p* = 0.000) backgrounds. Meanwhile, participants with the yellow background made lower errors than the reference white (*p* = 0.012), red (*p* = 0.002), blue (*p* = 0.002), orange (*p* = 0.001), and purple (*p* = 0.000) backgrounds (Table A3, Table A4 and Table A5).

Figure 3c visualises colour impacts on general performance in the Error-Speed space. Looking at error rate and response time together, participants were slower to respond, and their error rate was relatively higher with the purple and blue backgrounds, while participants reacted faster, and their error rate was significantly lower with the green background. These findings suggested that participants experienced a LA state when they completed questions with the purple and blue backgrounds and a HA state with the green background. Moreover, for the orange and red backgrounds, participants reacted significantly faster than with the purple, and they made slightly fewer errors than with the purple background. This suggested that participants experienced a HI state with the red and orange backgrounds. Regarding the yellow background, participants were shown to respond slower and made fewer errors, suggesting that participants experienced a LI state here.

### 3.2. Logical Abilities

**Figure 4:**
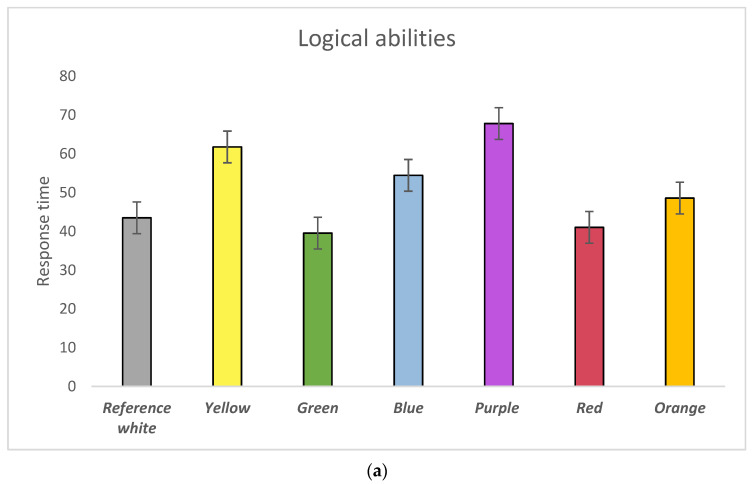

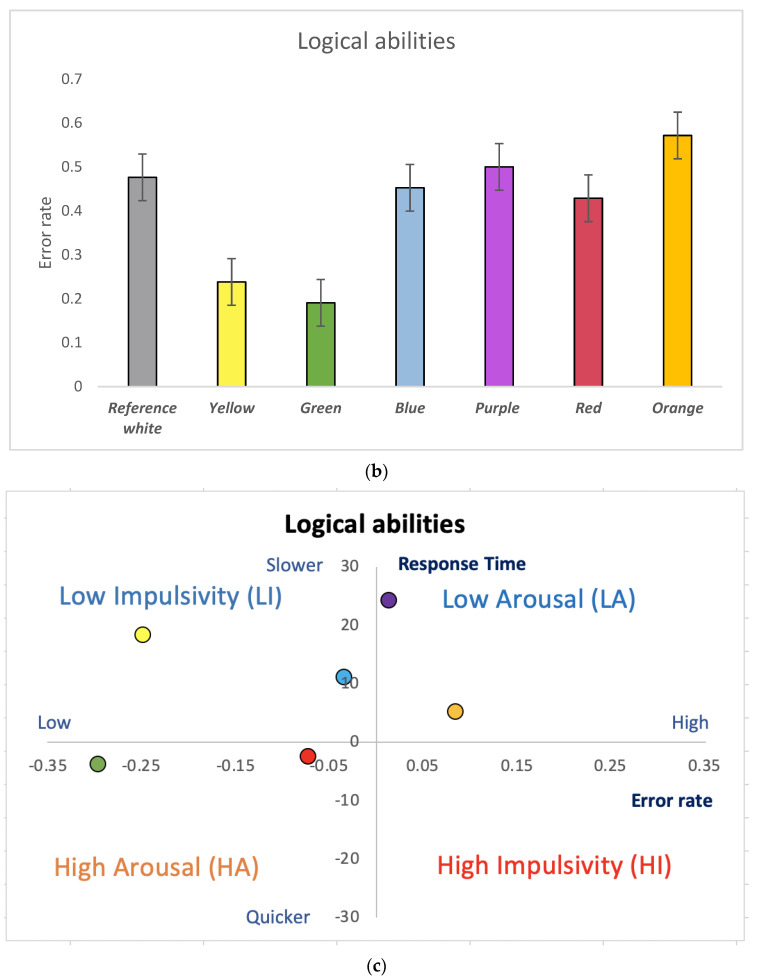
(**a**) Response time of participants’ performance in logical abilities by background colours; (**b**) Error rate of participants’ performance in logical abilities by background colours; (**c**) Colour impacts on logical abilities visualised in the Error-Speed space. The bars represent mean changes, while the error bars are the standard error of the mean across individual participants.

Participants’ logical abilities were validated by a logical rule test and mathematics sequence test. Generally, participants responded slowly with the purple background, but faster with the green. However, no statistical significance was observed in their response time with respect to colour influence on logical abilities (Figure 4a). Interestingly, we found participants’ logical abilities were significantly affected by colours with respect to their error rate (Figure 4b). Specifically, participants were shown to make significantly more errors with the orange background compared with the yellow (*p* = 0.002) and green (*p* = 0.000) backgrounds. Moreover, compared with the purple background, the participants’ error rate was significantly lower with the green (*p* = 0.000) and yellow (*p* = 0.002) backgrounds. Compared with the yellow, the participants’ error rate was shown to be higher than with the blue (*p* = 0.042) and reference white (*p* = 0.024) backgrounds. Furthermore, we observed that participants made fewer errors with the green (*p* = 0.003) compared with the red background (*p* = 0.024) and the reference white condition (*p* = 0.007) (Table A6, Table A7 and Table A8).

Together with both the error rate and response time (Figure 4c), our results suggested that participants’ logical abilities can be significantly influenced by green and red with an increasing aroused state and low aroused state with purple and orange. Meanwhile, blue and yellow were demonstrated to have low impulsive effects on participants’ logical abilities.

### 3.3. Lateral Abilities

**Figure 5:**
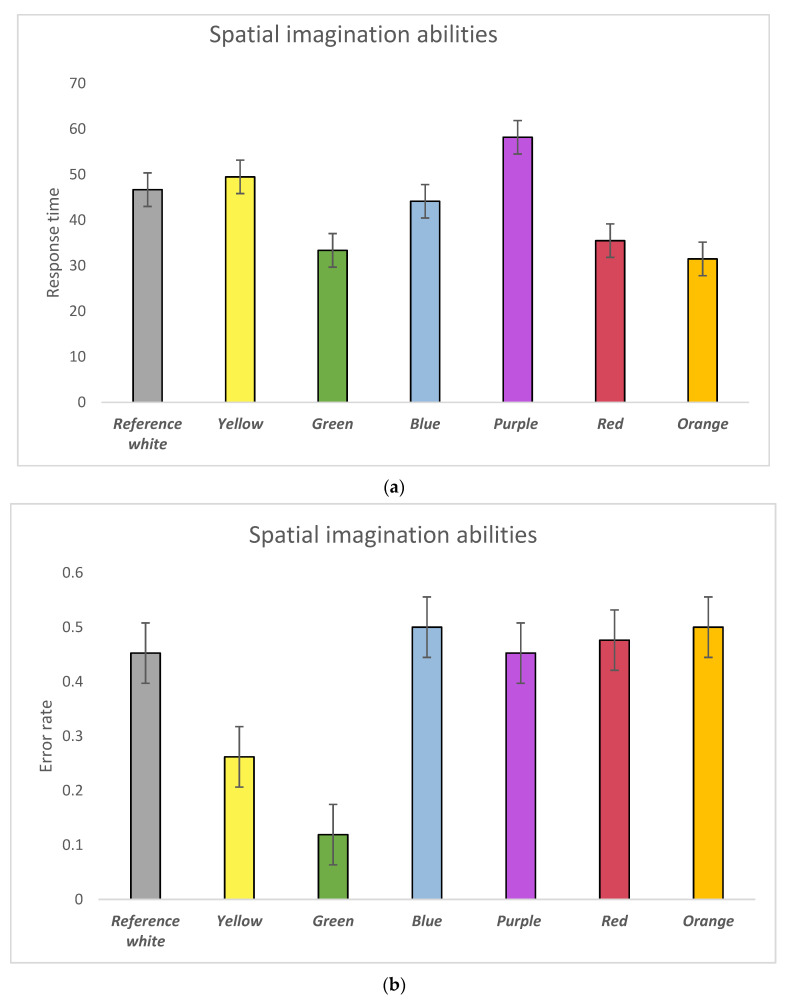

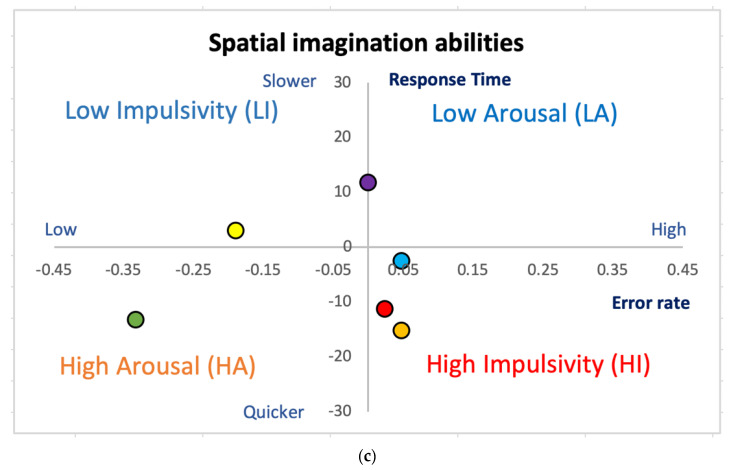
(**a**) Response time of participants’ performance in spatial imagination abilities by background colours; (**b**) Error rate of participants’ performance in spatial imagination abilities by background colours; (**c**) Colour impacts on spatial imagination abilities visualised in the Error-Speed space. The bars represent mean changes, while the error bars are the standard error of the mean across individual participants.

Results of the participants’ performance in relation to spatial imagination ability tests were shown to be significantly affected by colours with respect to their response time and error rate. Specifically, in terms of their response time (Figure 5a), participants reacted slower with the purple background compared with the red (*p* = 0.018) and green (*p* = 0.01) backgrounds. In addition, a significant difference was also observed between the orange and purple (*p* = 0.006) backgrounds. With regard to the error rate (Figure 5b), participants made fewer errors with the green background compared with the orange (*p* = 0.000), red (*p* = 0.001), purple (*p* = 0.001), blue (*p* = 0.000), and reference white condition (*p* = 0.000). Moreover, participants with the yellow background were shown to make fewer errors in lateral ability tests than those with the orange (*p* = 0.022), red (*p* = 0.040), and blue (*p* = 0.022) (Table A6, Table A7 and Table A8). Results of both error rate and response time (Figure 5c) of the spatial imagination ability tests suggested that participants experienced a HI state with orange, red, and blue backgrounds. Meanwhile, those with the green background were shown to be in a HA state, and the yellow background rarely induced a LI state.

### 3.4. Detail Abilities

**Figure 6:**
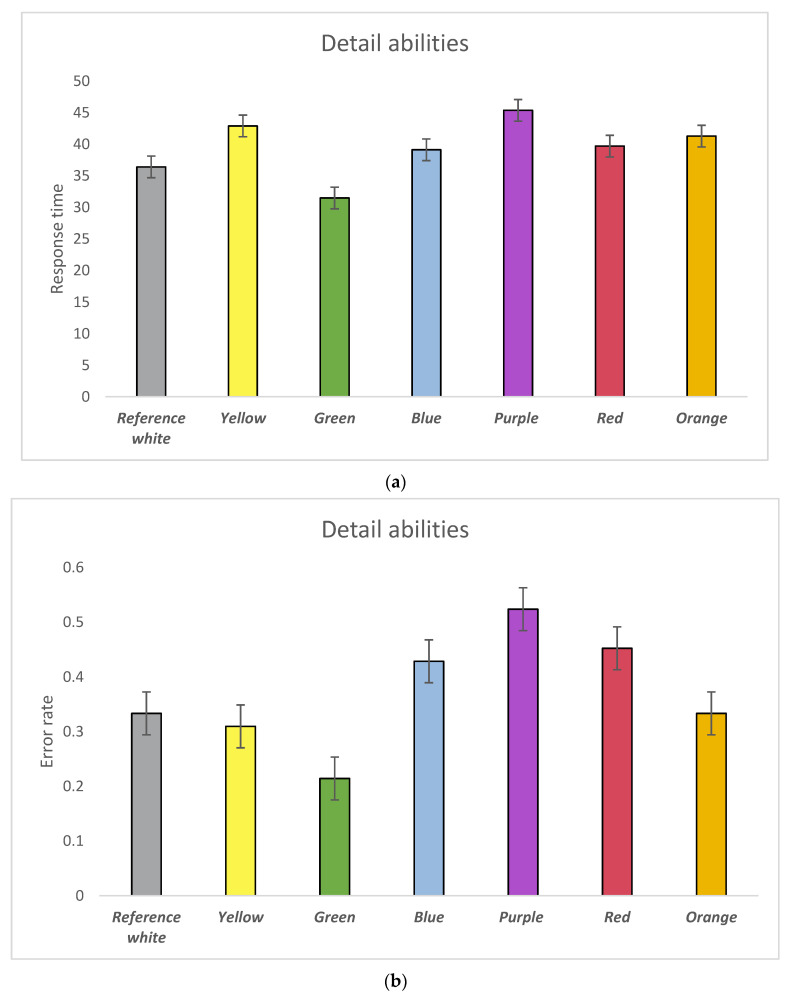

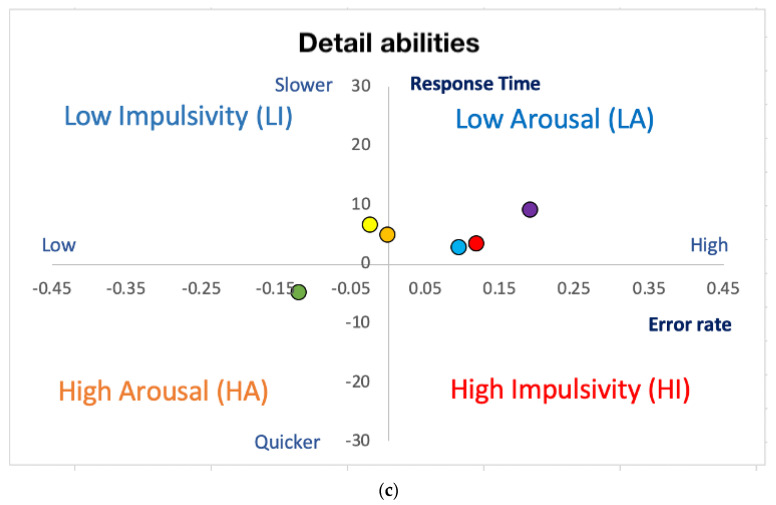
(**a**) Response time of participants’ performance in detail abilities by background colours; (**b**) Error rate of participants’ performance in detail abilities by background colours; (**c**) Colour impacts on detail imagination abilities visualised in the Error-Speed space. The bars represent mean changes, while the error bars are the standard error of the mean across individual participants.

Colour influence on detail abilities was validated through an odd one test and same detail test. Statistical significances were found in participants error rate. As shown in Figure 6a, participants reacted slower with the purple background than the green. However, no significant difference between these two colours on response time was found. Moreover, Figure 6b indicates that participants with the purple background made more errors than the green (*p* = 0.003) and yellow (*p* = 0.041) backgrounds. Meanwhile, participants made fewer errors with the green backgrounds than the blue (*p* = 0.041) and red (*p* = 0.031) backgrounds. Together with response time and error rate (Figure 6c), we found purple, red, and blue have LA effects on detail abilities. In addition, participants experienced a HA state with the green background, and rarely LI with the yellow background. Orange is located on the border between the LI and LA quadrants, while it is not the colour having no effects on detail abilities.

## 4. Discussion

This study explores the design potential of colour stimuli on cognitive abilities with a particular focus on people’s logical and lateral functions. Results from psychological experiments showed that colours can significantly influence people’s arousal and impulsiveness, suggesting that colour has indirect impacts on cognitive abilities. Specifically, findings concerning the colour impacts on general, logical, and spatial imagination, and detail abilities can be summarised as follows:

### 4.1. General Trend

- Purple leads to the lowest aroused state. It induced participants to make the most errors and had the longest reaction time.
- Green leads to the greatest aroused state. It induced participants to make the fewest errors and had the shortest reaction time.
- Yellow leads to the least impulsive state. Participants with yellow made the second most errors, while they reacted faster compared with green.
- Red and yellow are colours that influence people to be more impulsive.
- Blue seems to have a low aroused influence on participants’ performance. Participants with blue made more errors compared with orange, yellow, and green. Meanwhile, participants responded slower with blue compared with green.

### 4.2. Logical Abilities

- Colour seems to have no significant impact on participants’ reaction time on their logical performance.
- Yellow leads to the least impulsive state on participants’ logical performance.
- Yellow and green induced participants to make fewer errors in the logical ability test, suggesting green and yellow may have a positive impact on people’s logical abilities.
- Green and red are colours that influence people towards more arousal in logical performance.
- Red seems to have relatively high aroused effects on participants’ logical abilities.
- Purple and orange are colours that influence people towards low arousal in logical performance.
- Blue rarely has low impulsive impacts on logical abilities. It induced participants to make more errors in logical tests compared with yellow and green.

### 4.3. Spatial Imagination Abilities

- Green leads to the highest aroused state on spatial imagination abilities, suggesting green can positively stimulate people’s left cerebral hemisphere functions (lateral functions).
- Orange leads to the greatest impulsivity on lateral functions.
- Orange, red and blue seem to influence participants’ lateral functions with a high impulsivity state.
- Purple induced participants to make the most errors and had the longest reaction time in lateral ability tests.
- Red seems to have high impulsivity on participants’ lateral abilities, while it has relatively high aroused effects on participants’ logical abilities. Specifically, participants seem to make fewer errors in logical ability tests than lateral ability tests.
- Yellow has a low impulsive influence on participants’ lateral abilities.

### 4.4. Detail Abilities

- Purple leads to the lowest aroused state on participants’ detail abilities, suggesting purple has a relatively negative influence on people’s logical and lateral abilities.
- Green leads to the highest aroused influence on participants’ spatial imagination abilities. This also suggests that the colour green can positively influence people’s logical and lateral abilities.
- Purple, red, and blue are colours that have low aroused effects on detail abilities.
- Yellow and orange seem to have a relatively low impulsivity state on participants’ detail abilities. Specifically, participants made fewer errors with the orange background compared with the purple.

Above all, many studies have observed our findings and agreed that reddish colours (i.e., red, orange) can influence people with a high impulsivity state [11,33,43,44]. Moreover, we found green seems to have high aroused effects, which is consistent with Ciccone [45] whose results conflict with the conventional opinion that long wavelength (i.e., red light) lights are encouraging and short wavelength lights (i.e., blue light) are calming. In addition, our findings show that blue and yellow induced participants to make more errors, in agreement with Duan, Rhodes and Cheung [26]. However, our findings indicate that green seems to have high aroused effects and purple leads to the lowest aroused state, differing from Duan, Rhodes and Cheung [26] who found purple located in the high aroused quadrant and green seeming to have low aroused effects. A possible explanation for this could be that all participants involved in this study were animators (people good at lateral thinking), and this suggests that colours might have different impacts on lateral and logical thinkers. In that case, the participant selection criteria, although designed to ensure consistency, could be considered a study limitation.

## 5. Conclusions

The purpose of this study was to explore the influence of environmental colour on people’s logical and lateral abilities. This research used a psychological method to validate the impacts of colour on people’s response time and error rate in completing six types of psychometric tests (varied in hue backgrounds). Through the experiments, we found people’s logical and lateral functions can be significantly influenced by colours. Deliverable potentials of this work would add value to ongoing environmental design research, suggesting that researchers and designers should consider using colour to prompt people’s lateral and logical abilities. These experiments also retain certain limitations. First, due to the practical difficulties in conducting the study (each participant spent about 40 min), we included 21 participants, which is a relatively small number but nevertheless sufficient to show some significant results. Second, all participants were aged from 20 to 25, and thus the findings might not be generalisable to children and the elderly. Further experiments will be performed in the future to expand our findings.

## Data Availability

The data presented in this study are available on request from the corresponding author.

## Appendix A

**Table A1:** The Characteristics of the Background Colours.

Colours	L *	C *	h	a *	b *	R	G	B
Visual Reference White	70.01	0.51	28.34	0.24	0.19	171.27	170.01	169.86
Red	69.42	69.08	34.33	23.95	35.01	244.31	121.54	103.56
Yellow	70.52	69.13	99.63	−25.59	55.21	187.82	175.99	19.87
Blue	69.77	65.86	286.26	33.33	−36.64	110.09	158.86	255.00
Green	67.89	67.26	177.63	−54.76	−3.48	62.76	193.52	156.59
Orange	68.29	67.66	67.84	−0.57	56.18	242.31	154.31	55.88
Purple	68.01	68.17	320.95	48.07	−22.74	221.76	129.76	243.86

**Table A2:** Functions of Six Types of Psychometric Tests Used in the Experiments.

Cerebral Hemisphere	Cognitive Functions	Tests
Right cerebral hemisphere	Logical function	Logical abilities: Logical rule test Mathematics sequence
Left cerebral hemisphere	Lateral function	Spatial imagination abilities: Spatial structure test Rotation test
Right cerebral hemisphere: holistic perception; Left Right cerebral hemisphere: detail oriented perception	Logical & Lateral functions	Detail abilities: Odd one out Same detail test

**Table A3:** MANOVO Analysis of People’s Responses to Colours—General Effects.

Descriptive Statistics				
	Coloured_Backgrounds	Mean	Std. Deviation	*N*
Response_time	Reference white	42.1864	45.30772	126
	Red	38.7156	37.01419	126
	Yellow	51.4849	84.10959	126
	Blue	45.9029	49.64691	126
	Green	34.8059	30.16076	126
	Orange	40.4606	45.95477	126
	Purple	57.1974	73.77608	126
	Total	44.3934	55.60318	882
Error_rate	Reference white	0.4206	0.49563	126
	Red	0.4524	0.49971	126
	Yellow	0.2698	0.44565	126
	Blue	0.4603	0.50041	126
	Green	0.1746	0.38114	126
	Orange	0.4683	0.50098	126
	Purple	0.4921	0.50193	126
	Total	0.3912	0.48829	882

**Table A4:** Multivariate Tests of People’s Responses to Colours—General Effects.

Multivariate Tests ^a^									
Effect		Value	F	Hypothesis ^df^	Error ^df^	Sig.	Partial Eta Squared	Noncent. Parameter	Observed Power ^d^
**Intercept**	Pillai’s Trace	0.556	547.626 ^b^	2	874	0	0.556	1095.252	1
	Wilks’ Lambda	0.444	547.626 ^b^	2	874	0	0.556	1095.252	1
	Hotelling’s Trace	1.253	547.626 ^b^	2	874	0	0.556	1095.252	1
	Roy’s Largest Root	1.253	547.626 ^b^	2	874	0	0.556	1095.252	1
**Coloured_backgrounds**	Pillai’s Trace	0.068	5.128	12	1750	0	0.034	61.537	1
	Wilks’ Lambda	0.933	5.153 ^b^	12	1748	0	0.034	61.833	1
	Hotelling’s Trace	0.071	5.177	12	1746	0	0.034	62.128	1
	Roy’s Largest Root	0.056	8.226 ^c^	6	875	0	0.053	49.356	1

^a^ Design: Intercept + Coloured_backgrounds. ^b^ Exact statistic. ^c^ The statistic is an upper bound on F that yields a lower bound on the significance level. ^d^ Computed using alpha = 0.05.

**Table A5:** Multiple Comparisons of People’s Responses to Colours—General Effects.

Multiple Comparisons							
LSD							
Dependent Variable	(I) Coloured_Backgrounds	(J) Coloured_Backgrounds	Mean Difference (I–J)	Std. Error	Sig.	95% Confidence Interval	
						Lower Bound	Upper Bound
Response_time	Reference white	Red	3.4708	6.97038	0.619	−10.2098	17.1514
		Yellow	−9.2984	6.97038	0.183	−22.9791	4.3822
		Blue	−3.7165	6.97038	0.594	−17.3971	9.9641
		Green	7.3806	6.97038	0.29	−6.3001	21.0612
		Orange	1.7258	6.97038	0.805	−11.9548	15.4064
		Purple	−15.0110 *	6.97038	0.032	−28.6916	−1.3304
	Red	Reference white	−3.4708	6.97038	0.619	−17.1514	10.2098
		Yellow	−12.7693	6.97038	0.067	−26.4499	0.9113
		Blue	−7.1874	6.97038	0.303	−20.868	6.4933
		Green	3.9097	6.97038	0.575	−9.7709	17.5903
		Orange	−1.745	6.97038	0.802	−15.4256	11.9356
		Purple	−18.4818 *	6.97038	0.008	−32.1624	−4.8012
	Yellow	Reference white	9.2984	6.97038	0.183	−4.3822	22.9791
		Red	12.7693	6.97038	0.067	−0.9113	26.4499
		Blue	5.5819	6.97038	0.423	−8.0987	19.2625
		Green	16.6790 *	6.97038	0.017	2.9984	30.3596
		Orange	11.0242	6.97038	0.114	−2.6564	24.7048
		Purple	−5.7125	6.97038	0.413	−19.3931	7.9681
	Blue	Reference white	3.7165	6.97038	0.594	−9.9641	17.3971
		Red	7.1874	6.97038	0.303	−6.4933	20.868
		Yellow	−5.5819	6.97038	0.423	−19.2625	8.0987
		Green	11.0971	6.97038	0.112	−2.5835	24.7777
		Orange	5.4423	6.97038	0.435	−8.2383	19.1229
		Purple	−11.2944	6.97038	0.106	−24.9751	2.3862
	Green	Reference white	−7.3806	6.97038	0.29	−21.0612	6.3001
		Red	−3.9097	6.97038	0.575	−17.5903	9.7709
		Yellow	−16.6790 *	6.97038	0.017	−30.3596	−2.9984
		Blue	−11.0971	6.97038	0.112	−24.7777	2.5835
		Orange	−5.6548	6.97038	0.417	−19.3354	8.0258
		Purple	−22.3915 *	6.97038	0.001	−36.0721	−8.7109
	Orange	Reference white	−1.7258	6.97038	0.805	−15.4064	11.9548
		Red	1.745	6.97038	0.802	−11.9356	15.4256
		Yellow	−11.0242	6.97038	0.114	−24.7048	2.6564
		Blue	−5.4423	6.97038	0.435	−19.1229	8.2383
		Green	5.6548	6.97038	0.417	−8.0258	19.3354
		Purple	−16.7368 *	6.97038	0.017	−30.4174	−3.0561
	Purple	Reference white	15.0110 *	6.97038	0.032	1.3304	28.6916
		Red	18.4818 *	6.97038	0.008	4.8012	32.1624
		Yellow	5.7125	6.97038	0.413	−7.9681	19.3931
		Blue	11.2944	6.97038	0.106	−2.3862	24.9751
		Green	22.3915 *	6.97038	0.001	8.7109	36.0721
		Orange	16.7368 *	6.97038	0.017	3.0561	30.4174
Error_rate	Reference white	Red	−0.0317	0.06009	0.597	−0.1497	0.0862
		Yellow	0.1508 *	0.06009	0.012	0.0328	0.2687
		Blue	−0.0397	0.06009	0.509	−0.1576	0.0783
		Green	0.2460 *	0.06009	0	0.1281	0.364
		Orange	−0.0476	0.06009	0.428	−0.1656	0.0703
		Purple	−0.0714	0.06009	0.235	−0.1894	0.0465
	Red	Reference white	0.0317	0.06009	0.597	−0.0862	0.1497
		Yellow	0.1825 *	0.06009	0.002	0.0646	0.3005
		Blue	−0.0079	0.06009	0.895	−0.1259	0.11
		Green	0.2778 *	0.06009	0	0.1598	0.3957
		Orange	−0.0159	0.06009	0.792	−0.1338	0.1021
		Purple	−0.0397	0.06009	0.509	−0.1576	0.0783
	Yellow	Reference white	−0.1508 *	0.06009	0.012	−0.2687	−0.0328
		Red	−0.1825 *	0.06009	0.002	−0.3005	−0.0646
		Blue	−0.1905 *	0.06009	0.002	−0.3084	−0.0725
		Green	0.0952	0.06009	0.113	−0.0227	0.2132
		Orange	−0.1984 *	0.06009	0.001	−0.3164	−0.0805
		Purple	−0.2222 *	0.06009	0	−0.3402	−0.1043
	Blue	Reference white	0.0397	0.06009	0.509	−0.0783	0.1576
		Red	0.0079	0.06009	0.895	−0.11	0.1259
		Yellow	0.1905 *	0.06009	0.002	0.0725	0.3084
		Green	0.2857 *	0.06009	0	0.1678	0.4037
		Orange	−0.0079	0.06009	0.895	−0.1259	0.11
		Purple	−0.0317	0.06009	0.597	−0.1497	0.0862
	Green	Reference white	−0.2460 *	0.06009	0	−0.364	−0.1281
		Red	−0.2778 *	0.06009	0	−0.3957	−0.1598
		Yellow	−0.0952	0.06009	0.113	−0.2132	0.0227
		Blue	−0.2857 *	0.06009	0	−0.4037	−0.1678
		Orange	−0.2937 *	0.06009	0	−0.4116	−0.1757
		Purple	−0.3175 *	0.06009	0	−0.4354	−0.1995
	Orange	Reference white	0.0476	0.06009	0.428	−0.0703	0.1656
		Red	0.0159	0.06009	0.792	−0.1021	0.1338
		Yellow	0.1984 *	0.06009	0.001	0.0805	0.3164
		Blue	0.0079	0.06009	0.895	−0.11	0.1259
		Green	0.2937 *	0.06009	0	0.1757	0.4116
		Purple	−0.0238	0.06009	0.692	−0.1418	0.0941
	Purple	Reference white	0.0714	0.06009	0.235	−0.0465	0.1894
		Red	0.0397	0.06009	0.509	−0.0783	0.1576
		Yellow	0.2222 *	0.06009	0	0.1043	0.3402
		Blue	0.0317	0.06009	0.597	−0.0862	0.1497
		Green	0.3175 *	0.06009	0	0.1995	0.4354
		Orange	0.0238	0.06009	0.692	−0.0941	0.1418

Based on observed means. The error term is Mean Square (Error) = 0.228. * The mean difference is significant at the 0.05 level.

**Table A6:** MANOVO Analysis of People’s Logical, Lateral, and Detail Abilities Affected by Colours.

Descriptive Statistics				
	Coloured_Backgrounds	Mean	Std. Deviation	*N*
Logical_Response_Time	Reference white	43.462	36.98222	42
	Yellow	61.7532	129.30801	42
	Green	39.5396	37.95461	42
	Blue	54.4412	56.83899	42
	Purple	67.7787	105.389	42
	Red	41.0226	29.54236	42
	Orange	48.5597	54.56498	42
	Total	50.9367	73.33785	294
Logical_Error_rate	Reference white	0.4762	0.50549	42
	Yellow	0.2381	0.43108	42
	Green	0.1905	0.39744	42
	Blue	0.4524	0.50376	42
	Purple	0.5	0.50606	42
	Red	0.4286	0.50087	42
	Orange	0.5714	0.50087	42
	Total	0.4082	0.49233	294
Lateral_Response_Time	Reference white	46.6805	58.013	42
	Yellow	49.7937	48.91309	42
	Green	33.3861	28.51248	42
	Blue	44.1349	48.82134	42
	Purple	58.4328	57.48352	42
	Red	35.4139	28.68746	42
	Orange	31.5281	28.2063	42
	Total	42.7671	45.0009	294
Lateral_Error_rate	Reference white	0.4524	0.50376	42
	Yellow	0.2619	0.445	42
	Green	0.119	0.32777	42
	Blue	0.5	0.50606	42
	Purple	0.4524	0.50376	42
	Red	0.4762	0.50549	42
	Orange	0.5	0.50606	42
	Total	0.3946	0.48959	294
Detail_Response_Time	Reference white	36.4168	38.32565	42
	Yellow	42.9077	47.63413	42
	Green	31.4918	21.99098	42
	Blue	39.1327	42.13849	42
	Purple	45.3806	43.86046	42
	Red	39.7102	49.63497	42
	Orange	41.294	50.15938	42
	Total	39.4763	42.69481	294
Detail_Error_Rate	Reference white	0.3333	0.47712	42
	Yellow	0.3095	0.4679	42
	Green	0.2143	0.4153	42
	Blue	0.4286	0.50087	42
	Purple	0.5238	0.50549	42
	Red	0.4524	0.50376	42
	Orange	0.3333	0.47712	42
	Total	0.3707	0.48383	294

**Table A7:** Multivariate Tests of People’s Logical, Lateral, and Detail Abilities Affected by Colours.

Multivariate Tests ^a^									
Effect		Value	F	Hypothesis ^df^	Error ^df^	Sig.	Partial Eta Squared	Noncent. Parameter	Observed Power ^d^
Intercept	Pillai’s Trace	0.801	189.496 ^b^	6	282	0	0.801	1136.976	1
	Wilks’ Lambda	0.199	189.496 ^b^	6	282	0	0.801	1136.976	1
	Hotelling’s Trace	4.032	189.496 ^b^	6	282	0	0.801	1136.976	1
	Roy’s Largest Root	4.032	189.496 ^b^	6	282	0	0.801	1136.976	1
Coloured_backgrounds	Pillai’s Trace	0.235	1.952	36	1722	0.001	0.039	70.262	1
	Wilks’ Lambda	0.778	2.024	36	1241.11	0	0.041	52.907	0.994
	Hotelling’s Trace	0.267	2.082	36	1682	0	0.043	74.967	1
	Roy’s Largest Root	0.185	8.851 ^c^	6	287	0	0.156	53.108	1

^a^ Design: Intercept + Coloured_backgrounds. ^b^ Exact statistic. ^c^ The statistic is an upper bound on F that yields a lower bound on the significance level. ^d^ Computed using alpha = 0.05.

**Table A8:** Multiple Comparisons of People’s Logical, Lateral, and Detail Abilities Affected by Colours.

Multiple Comparisons							
LSD							
Dependent Variable	(I) Coloured_Backgrounds	(J) Coloured_Backgrounds	Mean Difference (I–J)	Std. Error	Sig.	95% Confidence Interval	
						Lower Bound	Upper Bound
Logical_Response_Time	Reference white	Yellow	−18.2911	16.01793	0.254	−49.8187	13.2364
		Green	3.9224	16.01793	0.807	−27.6051	35.4499
		Blue	−10.9792	16.01793	0.494	−42.5067	20.5483
		Purple	−24.3167	16.01793	0.13	−55.8442	7.2108
		Red	2.4395	16.01793	0.879	−29.0881	33.967
		Orange	−5.0977	16.01793	0.751	−36.6252	26.4298
	Yellow	Reference white	18.2911	16.01793	0.254	−13.2364	49.8187
		Green	22.2136	16.01793	0.167	−9.314	53.7411
		Blue	7.312	16.01793	0.648	−24.2155	38.8395
		Purple	−6.0256	16.01793	0.707	−37.5531	25.502
		Red	20.7306	16.01793	0.197	−10.7969	52.2581
		Orange	13.1934	16.01793	0.411	−18.3341	44.721
	Green	Reference white	−3.9224	16.01793	0.807	−35.4499	27.6051
		Yellow	−22.2136	16.01793	0.167	−53.7411	9.314
		Blue	−14.9016	16.01793	0.353	−46.4291	16.6259
		Purple	−28.2391	16.01793	0.079	−59.7666	3.2884
		Red	−1.483	16.01793	0.926	−33.0105	30.0446
		Orange	−9.0201	16.01793	0.574	−40.5476	22.5074
	Blue	Reference white	10.9792	16.01793	0.494	−20.5483	42.5067
		Yellow	−7.312	16.01793	0.648	−38.8395	24.2155
		Green	14.9016	16.01793	0.353	−16.6259	46.4291
		Purple	−13.3375	16.01793	0.406	−44.8651	18.19
		Red	13.4186	16.01793	0.403	−18.1089	44.9461
		Orange	5.8815	16.01793	0.714	−25.6461	37.409
	Purple	Reference white	24.3167	16.01793	0.13	−7.2108	55.8442
		Yellow	6.0256	16.01793	0.707	−25.502	37.5531
		Green	28.2391	16.01793	0.079	−3.2884	59.7666
		Blue	13.3375	16.01793	0.406	−18.19	44.8651
		Red	26.7562	16.01793	0.096	−4.7713	58.2837
		Orange	19.219	16.01793	0.231	−12.3085	50.7465
	Red	Reference white	−2.4395	16.01793	0.879	−33.967	29.0881
		Yellow	−20.7306	16.01793	0.197	−52.2581	10.7969
		Green	1.483	16.01793	0.926	−30.0446	33.0105
		Blue	−13.4186	16.01793	0.403	−44.9461	18.1089
		Purple	−26.7562	16.01793	0.096	−58.2837	4.7713
		Orange	−7.5372	16.01793	0.638	−39.0647	23.9904
	Orange	Reference white	5.0977	16.01793	0.751	−26.4298	36.6252
		Yellow	−13.1934	16.01793	0.411	−44.721	18.3341
		Green	9.0201	16.01793	0.574	−22.5074	40.5476
		Blue	−5.8815	16.01793	0.714	−37.409	25.6461
		Purple	−19.219	16.01793	0.231	−50.7465	12.3085
		Red	7.5372	16.01793	0.638	−23.9904	39.0647
Logical_Error_rate	Reference white	Yellow	0.2381 *	0.10468	0.024	0.0321	0.4441
		Green	0.2857 *	0.10468	0.007	0.0797	0.4918
		Blue	0.0238	0.10468	0.82	−0.1822	0.2299
		Purple	−0.0238	0.10468	0.82	−0.2299	0.1822
		Red	0.0476	0.10468	0.65	−0.1584	0.2537
		Orange	−0.0952	0.10468	0.364	−0.3013	0.1108
	Yellow	Reference white	−0.2381 *	0.10468	0.024	−0.4441	−0.0321
		Green	0.0476	0.10468	0.65	−0.1584	0.2537
		Blue	−0.2143 *	0.10468	0.042	−0.4203	−0.0082
		Purple	−0.2619 *	0.10468	0.013	−0.4679	−0.0559
		Red	−0.1905	0.10468	0.07	−0.3965	0.0156
		Orange	−0.3333 *	0.10468	0.002	−0.5394	−0.1273
	Green	Reference white	−0.2857 *	0.10468	0.007	−0.4918	−0.0797
		Yellow	−0.0476	0.10468	0.65	−0.2537	0.1584
		Blue	−0.2619 *	0.10468	0.013	−0.4679	−0.0559
		Purple	−0.3095 *	0.10468	0.003	−0.5156	−0.1035
		Red	−0.2381 *	0.10468	0.024	−0.4441	−0.0321
		Orange	−0.3810 *	0.10468	0	−0.587	−0.1749
	Blue	Reference white	−0.0238	0.10468	0.82	−0.2299	0.1822
		Yellow	0.2143 *	0.10468	0.042	0.0082	0.4203
		Green	0.2619 *	0.10468	0.013	0.0559	0.4679
		Purple	−0.0476	0.10468	0.65	−0.2537	0.1584
		Red	0.0238	0.10468	0.82	−0.1822	0.2299
		Orange	−0.119	0.10468	0.256	−0.3251	0.087
	Purple	Reference white	0.0238	0.10468	0.82	−0.1822	0.2299
		Yellow	0.2619 *	0.10468	0.013	0.0559	0.4679
		Green	0.3095 *	0.10468	0.003	0.1035	0.5156
		Blue	0.0476	0.10468	0.65	−0.1584	0.2537
		Red	0.0714	0.10468	0.496	−0.1346	0.2775
		Orange	−0.0714	0.10468	0.496	−0.2775	0.1346
	Red	Reference white	−0.0476	0.10468	0.65	−0.2537	0.1584
		Yellow	0.1905	0.10468	0.07	−0.0156	0.3965
		Green	0.2381 *	0.10468	0.024	0.0321	0.4441
		Blue	−0.0238	0.10468	0.82	−0.2299	0.1822
		Purple	−0.0714	0.10468	0.496	−0.2775	0.1346
		Orange	−0.1429	0.10468	0.173	−0.3489	0.0632
	Orange	Reference white	0.0952	0.10468	0.364	−0.1108	0.3013
		Yellow	0.3333 *	0.10468	0.002	0.1273	0.5394
		Green	0.3810 *	0.10468	0	0.1749	0.587
		Blue	0.119	0.10468	0.256	−0.087	0.3251
		Purple	0.0714	0.10468	0.496	−0.1346	0.2775
		Red	0.1429	0.10468	0.173	−0.0632	0.3489
Lateral_Response_Time	Reference white	Yellow	−3.1132	9.71618	0.749	−22.2372	16.0108
		Green	13.2943	9.71618	0.172	−5.8297	32.4183
		Blue	2.5455	9.71618	0.794	−16.5785	21.6695
		Purple	−11.7523	9.71618	0.227	−30.8763	7.3717
		Red	11.2665	9.71618	0.247	−7.8575	30.3905
		Orange	15.1524	9.71618	0.12	−3.9716	34.2764
	Yellow	Reference white	3.1132	9.71618	0.749	−16.0108	22.2372
		Green	16.4076	9.71618	0.092	−2.7165	35.5316
		Blue	5.6588	9.71618	0.561	−13.4652	24.7828
		Purple	−8.6391	9.71618	0.375	−27.7631	10.4849
		Red	14.3797	9.71618	0.14	−4.7443	33.5037
		Orange	18.2656	9.71618	0.061	−0.8584	37.3896
	Green	Reference white	−13.2943	9.71618	0.172	−32.4183	5.8297
		Yellow	−16.4076	9.71618	0.092	−35.5316	2.7165
		Blue	−10.7488	9.71618	0.27	−29.8728	8.3752
		Purple	−25.0466 *	9.71618	0.01	−44.1707	−5.9226
		Red	−2.0278	9.71618	0.835	−21.1518	17.0962
		Orange	1.8581	9.71618	0.848	−17.2659	20.9821
	Blue	Reference white	−2.5455	9.71618	0.794	−21.6695	16.5785
		Yellow	−5.6588	9.71618	0.561	−24.7828	13.4652
		Green	10.7488	9.71618	0.27	−8.3752	29.8728
		Purple	−14.2979	9.71618	0.142	−33.4219	4.8261
		Red	8.721	9.71618	0.37	−10.403	27.845
		Orange	12.6068	9.71618	0.195	−6.5172	31.7308
	Purple	Reference white	11.7523	9.71618	0.227	−7.3717	30.8763
		Yellow	8.6391	9.71618	0.375	−10.4849	27.7631
		Green	25.0466 *	9.71618	0.01	5.9226	44.1707
		Blue	14.2979	9.71618	0.142	−4.8261	33.4219
		Red	23.0188 *	9.71618	0.018	3.8948	42.1428
		Orange	26.9047 *	9.71618	0.006	7.7807	46.0287
	Red	Reference white	−11.2665	9.71618	0.247	−30.3905	7.8575
		Yellow	−14.3797	9.71618	0.14	−33.5037	4.7443
		Green	2.0278	9.71618	0.835	−17.0962	21.1518
		Blue	−8.721	9.71618	0.37	−27.845	10.403
		Purple	−23.0188 *	9.71618	0.018	−42.1428	−3.8948
		Orange	3.8859	9.71618	0.69	−15.2381	23.0099
	Orange	Reference white	−15.1524	9.71618	0.12	−34.2764	3.9716
		Yellow	−18.2656	9.71618	0.061	−37.3896	0.8584
		Green	−1.8581	9.71618	0.848	−20.9821	17.2659
		Blue	−12.6068	9.71618	0.195	−31.7308	6.5172
		Purple	−26.9047 *	9.71618	0.006	−46.0287	−7.7807
		Red	−3.8859	9.71618	0.69	−23.0099	15.2381
Lateral_Error_rate	Reference white	Yellow	0.1905	0.1037	0.067	−0.0136	0.3946
		Green	0.3333 *	0.1037	0.001	0.1292	0.5374
		Blue	−0.0476	0.1037	0.646	−0.2517	0.1565
		Purple	0	0.1037	1	−0.2041	0.2041
		Red	−0.0238	0.1037	0.819	−0.2279	0.1803
		Orange	−0.0476	0.1037	0.646	−0.2517	0.1565
	Yellow	Reference white	−0.1905	0.1037	0.067	−0.3946	0.0136
		Green	0.1429	0.1037	0.169	−0.0612	0.347
		Blue	−0.2381 *	0.1037	0.022	−0.4422	−0.034
		Purple	−0.1905	0.1037	0.067	−0.3946	0.0136
		Red	−0.2143 *	0.1037	0.04	−0.4184	−0.0102
		Orange	−0.2381 *	0.1037	0.022	−0.4422	−0.034
	Green	Reference white	−0.3333 *	0.1037	0.001	−0.5374	−0.1292
		Yellow	−0.1429	0.1037	0.169	−0.347	0.0612
		Blue	−0.3810 *	0.1037	0	−0.5851	−0.1768
		Purple	−0.3333 *	0.1037	0.001	−0.5374	−0.1292
		Red	−0.3571 *	0.1037	0.001	−0.5612	−0.153
		Orange	−0.3810 *	0.1037	0	−0.5851	−0.1768
	Blue	Reference white	0.0476	0.1037	0.646	−0.1565	0.2517
		Yellow	0.2381 *	0.1037	0.022	0.034	0.4422
		Green	0.3810 *	0.1037	0	0.1768	0.5851
		Purple	0.0476	0.1037	0.646	−0.1565	0.2517
		Red	0.0238	0.1037	0.819	−0.1803	0.2279
		Orange	0	0.1037	1	−0.2041	0.2041
	Purple	Reference white	0	0.1037	1	−0.2041	0.2041
		Yellow	0.1905	0.1037	0.067	−0.0136	0.3946
		Green	0.3333 *	0.1037	0.001	0.1292	0.5374
		Blue	−0.0476	0.1037	0.646	−0.2517	0.1565
		Red	−0.0238	0.1037	0.819	−0.2279	0.1803
		Orange	−0.0476	0.1037	0.646	−0.2517	0.1565
	Red	Reference white	0.0238	0.1037	0.819	−0.1803	0.2279
		Yellow	0.2143 *	0.1037	0.04	0.0102	0.4184
		Green	0.3571 *	0.1037	0.001	0.153	0.5612
		Blue	−0.0238	0.1037	0.819	−0.2279	0.1803
		Purple	0.0238	0.1037	0.819	−0.1803	0.2279
		Orange	−0.0238	0.1037	0.819	−0.2279	0.1803
	Orange	Reference white	0.0476	0.1037	0.646	−0.1565	0.2517
		Yellow	0.2381 *	0.1037	0.022	0.034	0.4422
		Green	0.3810 *	0.1037	0	0.1768	0.5851
		Blue	0	0.1037	1	−0.2041	0.2041
		Purple	0.0476	0.1037	0.646	−0.1565	0.2517
		Red	0.0238	0.1037	0.819	−0.1803	0.2279
Detail_Response_Time	Reference white	Yellow	−6.491	9.36793	0.489	−24.9295	11.9476
		Green	4.925	9.36793	0.599	−13.5136	23.3635
		Blue	−2.716	9.36793	0.772	−21.1545	15.7226
		Purple	−8.9639	9.36793	0.339	−27.4024	9.4747
		Red	−3.2935	9.36793	0.725	−21.7321	15.1451
		Orange	−4.8773	9.36793	0.603	−23.3159	13.5613
	Yellow	Reference white	6.491	9.36793	0.489	−11.9476	24.9295
		Green	11.4159	9.36793	0.224	−7.0227	29.8545
		Blue	3.775	9.36793	0.687	−14.6636	22.2136
		Purple	−2.4729	9.36793	0.792	−20.9115	15.9657
		Red	3.1975	9.36793	0.733	−15.2411	21.636
		Orange	1.6137	9.36793	0.863	−16.8249	20.0522
	Green	Reference white	−4.925	9.36793	0.599	−23.3635	13.5136
		Yellow	−11.4159	9.36793	0.224	−29.8545	7.0227
		Blue	−7.6409	9.36793	0.415	−26.0795	10.7977
		Purple	−13.8888	9.36793	0.139	−32.3274	4.5498
		Red	−8.2185	9.36793	0.381	−26.657	10.2201
		Orange	−9.8023	9.36793	0.296	−28.2408	8.6363
	Blue	Reference white	2.716	9.36793	0.772	−15.7226	21.1545
		Yellow	−3.775	9.36793	0.687	−22.2136	14.6636
		Green	7.6409	9.36793	0.415	−10.7977	26.0795
		Purple	−6.2479	9.36793	0.505	−24.6865	12.1907
		Red	−0.5775	9.36793	0.951	−19.0161	17.861
		Orange	−2.1613	9.36793	0.818	−20.5999	16.2772
	Purple	Reference white	8.9639	9.36793	0.339	−9.4747	27.4024
		Yellow	2.4729	9.36793	0.792	−15.9657	20.9115
		Green	13.8888	9.36793	0.139	−4.5498	32.3274
		Blue	6.2479	9.36793	0.505	−12.1907	24.6865
		Red	5.6704	9.36793	0.545	−12.7682	24.1089
		Orange	4.0866	9.36793	0.663	−14.352	22.5251
	Red	Reference white	3.2935	9.36793	0.725	−15.1451	21.7321
		Yellow	−3.1975	9.36793	0.733	−21.636	15.2411
		Green	8.2185	9.36793	0.381	−10.2201	26.657
		Blue	0.5775	9.36793	0.951	−17.861	19.0161
		Purple	−5.6704	9.36793	0.545	−24.1089	12.7682
		Orange	−1.5838	9.36793	0.866	−20.0224	16.8548
	Orange	Reference white	4.8773	9.36793	0.603	−13.5613	23.3159
		Yellow	−1.6137	9.36793	0.863	−20.0522	16.8249
		Green	9.8023	9.36793	0.296	−8.6363	28.2408
		Blue	2.1613	9.36793	0.818	−16.2772	20.5999
		Purple	−4.0866	9.36793	0.663	−22.5251	14.352
		Red	1.5838	9.36793	0.866	−16.8548	20.0224
Detail_Error_Rate	Reference white	Yellow	0.0238	0.10455	0.82	−0.182	0.2296
		Green	0.119	0.10455	0.256	−0.0867	0.3248
		Blue	−0.0952	0.10455	0.363	−0.301	0.1105
		Purple	−0.1905	0.10455	0.07	−0.3963	0.0153
		Red	−0.119	0.10455	0.256	−0.3248	0.0867
		Orange	0	0.10455	1	−0.2058	0.2058
	Yellow	Reference white	−0.0238	0.10455	0.82	−0.2296	0.182
		Green	0.0952	0.10455	0.363	−0.1105	0.301
		Blue	−0.119	0.10455	0.256	−0.3248	0.0867
		Purple	−0.2143 *	0.10455	0.041	−0.4201	−0.0085
		Red	−0.1429	0.10455	0.173	−0.3486	0.0629
		Orange	−0.0238	0.10455	0.82	−0.2296	0.182
	Green	Reference white	−0.119	0.10455	0.256	−0.3248	0.0867
		Yellow	−0.0952	0.10455	0.363	−0.301	0.1105
		Blue	−0.2143 *	0.10455	0.041	−0.4201	−0.0085
		Purple	−0.3095 *	0.10455	0.003	−0.5153	−0.1037
		Red	−0.2381 *	0.10455	0.024	−0.4439	−0.0323
		Orange	−0.119	0.10455	0.256	−0.3248	0.0867
	Blue	Reference white	0.0952	0.10455	0.363	−0.1105	0.301
		Yellow	0.119	0.10455	0.256	−0.0867	0.3248
		Green	0.2143 *	0.10455	0.041	0.0085	0.4201
		Purple	−0.0952	0.10455	0.363	−0.301	0.1105
		Red	−0.0238	0.10455	0.82	−0.2296	0.182
		Orange	0.0952	0.10455	0.363	−0.1105	0.301
	Purple	Reference white	0.1905	0.10455	0.07	−0.0153	0.3963
		Yellow	0.2143 *	0.10455	0.041	0.0085	0.4201
		Green	0.3095 *	0.10455	0.003	0.1037	0.5153
		Blue	0.0952	0.10455	0.363	−0.1105	0.301
		Red	0.0714	0.10455	0.495	−0.1344	0.2772
		Orange	0.1905	0.10455	0.07	−0.0153	0.3963
	Red	Reference white	0.119	0.10455	0.256	−0.0867	0.3248
		Yellow	0.1429	0.10455	0.173	−0.0629	0.3486
		Green	0.2381 *	0.10455	0.024	0.0323	0.4439
		Blue	0.0238	0.10455	0.82	−0.182	0.2296
		Purple	−0.0714	0.10455	0.495	−0.2772	0.1344
		Orange	0.119	0.10455	0.256	−0.0867	0.3248
	Orange	Reference white	0	0.10455	1	−0.2058	0.2058
		Yellow	0.0238	0.10455	0.82	−0.182	0.2296
		Green	0.119	0.10455	0.256	−0.0867	0.3248
		Blue	−0.0952	0.10455	0.363	−0.301	0.1105
		Purple	−0.1905	0.10455	0.07	−0.3963	0.0153
		Red	−0.119	0.10455	0.256	−0.3248	0.0867

Based on observed means. The error term is Mean Square (Error) = 0.230. * The mean difference is significant at the 0.05 level.
